# Imbalance in mitochondrial dynamics induced by low PGC-1α expression contributes to hepatocyte EMT and liver fibrosis

**DOI:** 10.1038/s41419-020-2429-9

**Published:** 2020-04-08

**Authors:** Linzhong Zhang, Yanghao Zhang, Xinxiang Chang, Xiuying Zhang

**Affiliations:** Department of Histology and Embryology, School of Basic Medical Sciences, Capital Medical University, Beijing, China

**Keywords:** Liver fibrosis, Experimental models of disease

## Abstract

An imbalance in mitochondrial dynamics induced by oxidative stress may lead to hepatocyte epithelial mesenchymal transition (EMT) and liver fibrosis. However, the underlying molecular mechanisms have not been fully elucidated. This study investigated the role of mitochondrial dynamics in hepatocyte EMT and liver fibrosis using an in vitro human (L-02 cells, hepatic cell line) and an in vivo mouse model of liver fibrosis. Findings showed that oxidative stress-induced mitochondrial DNA damage was associated with abnormal mitochondrial fission and hepatocyte EMT. The reactive oxygen species (ROS) scavengers apocynin and mito-tempo effectively attenuated carbon tetrachloride (CCl_4_)-induced abnormal mitochondrial fission and liver fibrosis. Restoring mitochondrial biogenesis attenuated hepatocyte EMT. Oxidative stress-induced abnormal hepatocyte mitochondrial fission events by a mechanism that involved the down regulation of PGC-1α. PGC-1α knockout mice challenged with CCl_4_ had increased abnormal mitochondrial fission and more severe liver fibrosis than wild type mice. These results indicate that PGC-1α has a protective role in oxidative stress-induced-hepatocyte EMT and liver fibrosis.

## Introduction

Liver fibrosis occurs in many forms of liver disease, including hepatitis and chronic alcoholism, and results in scarring and damage to the liver^1–3^. Liver fibrosis is a wound-healing response that is characterized by excessive deposition of extracellular matrix (ECM) proteins, hepatocyte damage, distortion of the hepatic lobules, and changes in the vascular architecture^4^. If left untreated, liver fibrosis can progress to cirrhosis or hepatocellular carcinoma; therefore, elucidation of the mechanisms underlying liver fibrosis is crucial.

Overproduction of reactive oxygen species (ROS) results in oxidative stress, which is involved in the pathophysiology of cardiovascular disease, cancer, and aging. Oxidative stress causes macromolecular damage to cellular lipids, proteins, or DNA^5–9^. In particular, ROS cause damage and large-scale deletion of mitochondrial DNA. Our previous work in a rat model of renal ischemia-reperfusion injury revealed that oxidative stress-induced damage and deletion of mitochondrial DNA was associated with epithelial to mesenchymal transition (EMT)^10^.

Tightly regulated cycles of fusion and fission adapt mitochondrial morphology to the metabolic needs of a cell, such that mitochondrial fusion increases metabolic efficiency, while mitochondrial fission favors uncoupled respiration^11^. Mitochondrial fusion is stimulated by energy demand and stress. Stress-induced fusion allows mitochondria to share components and compensate for defect protein complexes or DNA sequences that are essential for ATP production. Mitochondrial fission usually occurs under relaxed conditions, when new mitochondria are generated and dysfunctional mitochondria are segregated to maintain a healthy mitochondrial network. Mitochondrial fission can also occur during high levels of cellular stress, when it activates apoptosis^12–14^. Mitochondrial dynamics, defined as mitochondrial fission, fusion and the localization of mitochondria within cells, may play an important role in the pathophysiology of liver fibrosis. We propose that hepatocyte EMT is associated with an oxidative stress-induced imbalance in mitochondrial dynamics. Thus, modulation of mitochondrial dynamics has potential to reverse EMT and liver fibrosis.

Mitochondrial dysfunction is a prominent feature of some diseases. Dynamin-related/-like protein 1 (Drp1) and dynamin2 (Dnm2), large GTPase proteins belonging to the dynamin family, are key components of the mitochondrial fission machinery. Dnm1/Drp1 are cytosolic proteins that are recruited to the mitochondrial surface by mitochondrial fission factor (MFF) or mitochondrial fission 1 (FIS1). Dnm1/Drp1 assemble on the surface of mitochondria and drive membrane constriction and the scission of the inner and outer mitochondrial membranes in a GTP-dependent manner. The endoplasmic reticulum (ER), ER-bound inverted-formin 2 (INF2), and actin assembly at mitochondria-ER contact sites are required for mitochondrial fission^15–17^. An increase in phospho-Drp1 levels was detected in mitochondrial outer-membrane and forms a large ring-like complex to exert fission activity in sporadic Parkinson’s disease cellular models, implicating increased mitochondrial fission in the pathophysiology of Parkinson’s diseases^18,19^. However, the role of mitochondrial dynamics and its potential as a therapeutic target in liver fibrosis remains to be elucidated.

Peroxisome proliferator-activated receptor gamma coactivator 1-alpha (PGC-1α) is a peroxisome proliferator-activated receptor (PPARγ) assisted transcription activator. PGC-1α promotes the expression of mitochondrial genes encoded in the nucleus by activating various transcription factors and nuclear hormone receptors^20^. PGC-1α may prevent EMT by improving mitochondrial dysfunction. In diabetic nephropathy, decreased PGC-1α was associated with increased expression of Drp1, mitochondrial fission, and suppressed inflammation^21^. To date, the role of PGC-1α in regulating mitochondrial dynamics, hepatocyte EMT, and liver fibrosis are unknown.

The objective of the current study was to assess the role of mitochondrial dynamics in hepatocyte EMT and liver fibrosis. Findings showed that oxidative stress-induced mitochondrial DNA damage was associated with abnormal mitochondrial fission and hepatocyte EMT. ROS scavengers effectively attenuated carbon tetrachloride (CCl_4_)-induced abnormal mitochondrial fission and liver fibrosis. Inhibiting abnormal mitochondrial fission attenuated hepatocyte EMT and liver fibrosis. Oxidative stress-induced abnormal hepatocyte mitochondrial fission events by a mechanism that involved the down regulation of PGC-1α. PGC-1α overexpression markedly reduced Drp1 protein levels and prevented hepatocyte EMT. Conversely, silencing PGC-1α increased Drp1 protein levels and hepatocyte EMT. PGC-1α knockout mice challenged with CCl_4_ had increased abnormal mitochondrial fission and more severe liver fibrosis than wild type mice.

## Methods

### Reagents

L-02 cells (human hepatic cell line) were purchased from ATCC (Manassas, VA, USA). Anti-Drp1, ani-COX IV, anti-E-cadherin, anti-collagen I, anti-GAPDH and anti-β-actin antibodies were purchased from Cell Signaling Technology (Beverly, MA, USA). Anti-PGC-1α, anti-TFIIB, and anti-MFN1 antibodies were obtained from Abcam (Cambridge, MA, USA). Anti-α-SMA antibody, mitochondria isolation kits, and Mdivi-1 were purchased from Sigma-Aldrich (St Louis, MO, USA). Anti-albumin antibody was purchased from Proteintech (Chicago, IL, USA). Anti-nitrotyrosine antibody and lipofectamine 2000 were purchased from Invitrogen (Carlsbad, CA, USA). FITC conjugated anti-rabbit IgG, cy3 conjugated anti-mouse IgG, and PGC-1α CRISPR activation plasmids were purchased from Santa Cruz Biotechnology (Santa Cruz, CA, USA). Drp1 and PGC-1α small interfering RNAs (siRNAs) (Table 1) were purchased from Oligobio (Beijing, China). Masson’s trichrome staining kits were purchased from Jiancheng Biological Engineering Institute (Nanjing, Jiangsu, China). Streptomycin-biotin-peroxidase immunohistochemical staining kits were purchased from Maixin Biological Technology Corporation (Fuzhou, Fujian, China). DNeasy and RNeasy blood and tissue kits were purchased from Qiagen (Dusseldorf, Germany).

**Table 1:** siRNA sequences.

siDrp1 (human) 5′-AAGCAGAAGAATGGGGTAAAT-3′
siPGC-1α (human) 5′-GUCGCAGUCACAACACUUATT-3′
NC5′-CAGUACUUUUGUGUAGUACAA-3′

*NC* Negative control.

### Animals and liver fibrosis models

Six-week-old male C57BL/6 mice were obtained from Beijing Vital River Laboratory Animal Technology Co., Ltd. (Beijing, China). Ppargc1a^f/f^ (B6.Cg-Ppargc1α^tm2.1Brsp^/J) and Tg (Alb-cre)^21Mgn^/J (Alb-cre^+/+^) mice were obtained from the Jackson Laboratory (Bar Harbor, ME). Hepatocyte-specific PGC-1α knockout mice were generated based on the classic Cre-loxP recombination system. Briefly, Alb-cre^+/+^ mice were crossed with C57BL/6 mice to generate Alb-cre^+/0^ mice. Alb-cre^+/0^ mice were crossed with Ppargc1α^f/f^ mice to generate Ppargc1α^f/+^Alb-cre^+/0^ mice. Alb-cre^0/0^ mice (Ppargc1α^+/+^) or hepatocyte-specific knockout mice (Ppargc1α^f/f^Alb-cre^+/0^) were selected by crossing Ppargc1α^f/+^Alb-cre^+/0^ and Ppargc1α^f/+^Alb-cre^+/0^mice. Mice were treated in accordance with the guidelines approved by the Animal Care Committee of Capital Medical University, which complies with the guidelines issued by the National Institutes of Health (NIH). The total number of mice used in the current study was approximately 180. Randomization was used to determine animals were allocated to experimental groups. The operator or investigator was blinded to the grouping. Fibrosis was induced in mice using carbon tetrachloride (CCl_4_) dissolved in olive oil (1:9) (1 ml/kg body weight twice weekly). Controls were administered an equal volume of olive oil vehicle. Liver tissues were collected at 2 weeks, 4 weeks, 6 weeks and 8 weeks. Liver tissues were fixed in 10% formaldehyde solution, embedded in paraffin, and sectioned to a thickness of 4μm, or stored in RNAlater, or snap frozen in liquid nitrogen and stored at −80 °C.

### Apocynin and mito-tempo treatment

**Fig. 1:**
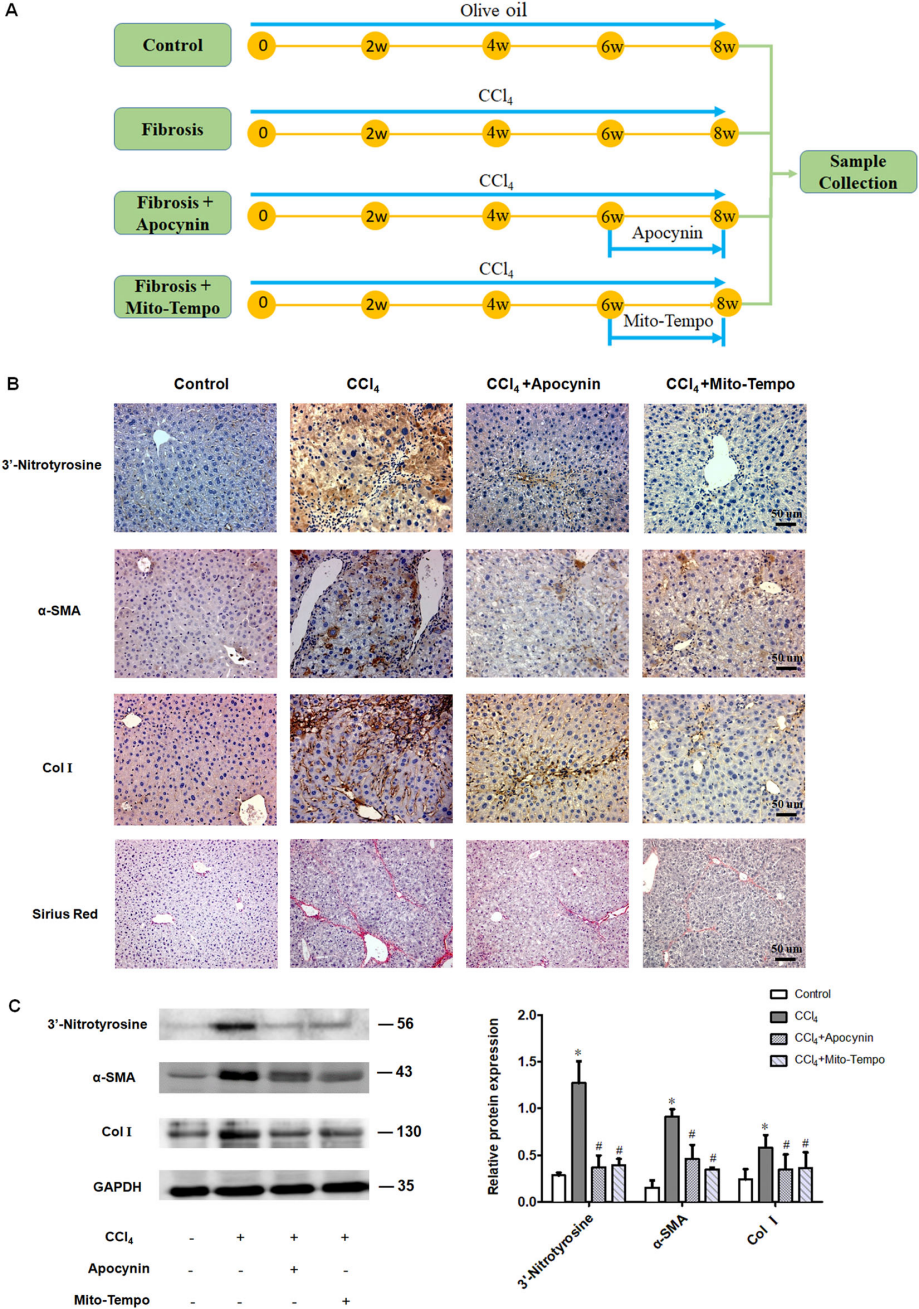
ROS scavengers attenuate the progression of liver fibrosis. **a** Apocynin and mito-tempo treatment protocol. **b** Immunohistochemistry showing 3-nitrotyrosine, α-SMA and collagen I expression were increased in liver tissue derived from mice challenged with CCl_4_ for 8 weeks. Treatment with apocynin and mito-tempo attenuated the CCl_4_-induced increases in 3′-nitrotyrosine, α-SMA and collagen I expression, and sirus red staining showed a markedly decreased number of fibrous septa (20×); **c** Western blot analysis showing treatment with apocynin and mito-tempo attenuated CCl_4_-induced increases in 3′-nitrotyrosine, α-SMA and collagen I protein levels. GAPDH served as the internal control. *n* = 4 mice in each group; experiments were repeated at least three times. The total number of animals in each group was approximately 12. *P < 0.05 vs. control, ^#^*P* < 0.05 vs. CCl_4_ group.

Mice were randomly assigned to four groups. Controls: mice were treated with olive oil vehicle twice a week for 8 weeks. Fibrosis: mice were treated with CCl_4_ (0.1 ml/kg body weight) twice a week for 8 weeks. Fibrosis + Apocynin: mice were treated with CCl_4_ twice a week for 8 weeks, and 10% DMSO containing apocynin (10 mg/kg) was injected intraperitoneally every other day during the last two weeks. Fibrosis + Mito-Tempo: mice were treated with CCl_4_ twice a week for 8 weeks, and 0.9% normal saline containing mito-tempo (10 mg/kg) was injected intraperitoneally every other day during the last 2 weeks (Fig. 1a).

### Mdivi-1 treatment

Mdivi-1 was dissolved in dimethyl sulfoxide (DMSO) to a final concentration of 8 mg/kg. Mice were divided into four groups. Controls: mice were treated with olive oil vehicle twice a week for 6 weeks. Fibrosis: mice were treated with CCl_4_ (0.1 ml/kg body weight) twice a week for 6 weeks. Fibrosis + DMSO: mice were treated with CCl_4_ twice a week for 6 weeks, and DMSO was injected intraperitoneally every other day during the last two weeks. Fibrosis + Mdivi-1: mice were treated with CCl_4_ twice a week for 6 weeks, and Mdivi-1 was injected intraperitoneally every other day during the last 2 weeks (Fig. 4a).

### Isolation of mitochondria DNA

Mitochondrial fractions were isolated from liver tissues and L-02 cells as previously described^10^. Mitochondrial DNA was extracted with a DNeasy blood and tissue kit according to the manufacturer’s instructions.

### Quantitative PCR analysis

Total RNA was extracted from liver tissues and L-02 cells with Trizol reagent according to the manufacturer’s instructions. Mitochondrial DNA depletion was evaluated as previously described^10,22^. Quantitative PCR used SYBR Green Master mix on the ABI prism 7500 detection system (Applied Biosystems, Foster City, CA) according to the manufacturer’s instructions. Primers for mitochondrial DNA amplification were 5′-CGCTCTACCTCACCATCTCTT-3′ and 5′-TGCTTACCTTGTTACGACTT-3′. Primers for PGC-1α amplification were 5′-TACAACAATGAGCCTGCGAAC-3′ and 5′-CATCAAATGAGGGCAATCCG-3′. Primers for GAPDH amplification were 5′-GCGACTTCAACAGCAACTCCC-3′ and 5′-TTGCTGTAGCCGTATTCATTGTCA-3′. Standardization was performed using GAPDH and evaluated with the comparative CT method (2-^ΔΔCT^).

### Western blot analysis

Proteins were extracted from liver tissues, liver mitochondria and L-02 cells with RIPA lysis buffer (50 mM Tris-HCl [pH 7.4], 150 mM NaCl, 1% NP-40, 0.1% SDS, protease inhibitor and phosphatase inhibitors). Proteins were separated by polyacrylamide SDS electrophoresis at 80 V for 0.5 h and 100 V for 2 h and electrophoretically transferred onto poly(vinylidene fluoride) membranes (Merck Millipore, Billerica, MA, USA) at 0.3 A for 2.5 h. Membranes were blocked with 5% non-fat milk or bovine serum albumin (BSA) and incubated with primary antibodies, including anti-α-SMA (1:3000), anti-Drp1 (1:5000), anti-COX IV (1:4000), anti-e-cadherin (1:2000), anti-collagen I (1:3000), anti-PGC-1α (1:2000), anti-MFN1 (1:3000), anti-albumin (1:3000), anti-3′-nitrotyrosine (1:1000), anti-HNF-4 (1:1000), anti-TFIIB (1:2000), anti-GAPDH (1:6000) or anti-β–actin (1:5000), overnight at 4 °C. Membranes were incubated with a horseradish peroxidase-conjugated secondary antibody for 1 h at room temperature. Protein bands were visualized using luminal chemiluminescence (Bio-Rad; Hercules, CA, USA). Gray scale values were analyzed using ImageJ software (NIH).

### Cell culture and transfection

L-02 cells were cultured in low glucose Dulbecco’s modified Eagle’s medium supplemented with 20% bovine fetal serum and 1% antibiotic (penicillin and streptomycin). Cultured cells were treated with 300 μM H_2_O_2_ and harvested at 6, 12, 24, 36, and 48 h. Cells were transfected with PGC-1α (accession code: NM-013261) siRNA, Drp1 (accession code: NM-012062) siRNA or a PGC-1α CRISPR activation plasmid using lipofectamine 2000, according to the manufacturer’s instructions. The transfection medium was removed after 8 h, and cells were treated with 300 μM H_2_O_2_ in complete medium for 48 h. Cells were harvested for analysis of PGC-1α, Drp1, albumin, e-cadherin, α-SMA, vimentin and fibronectin proteins.

### Immunofluorescence staining

Frozen liver sections were fixed in acetone for 10 min at −20 °C, treated with blocking buffer (5% normal donkey serum in 1% BSA with 0.3% Triton X-100), and incubated with anti-α-SMA antibody (diluted 1:1000 in 0.1% BSA) and anti Drp1 antibody (1:100), or anti-HNF-4 antibody (1:200) and anti-PGC-1α antibody (1:300) overnight at 4 °C. Then, sections were incubated with FITC donkey anti-mouse IgG (1:300) and Cy3-labeled donkey anti-rabbit IgG (1:300) antibodies at room temperature for 1 h. Cells on slides were fixed in 4% paraformaldehyde for 20 min, permeabilized with 0.3% Triton X-100 for 30 min, and incubated with anti-α-SMA antibody (1:1000) and anti-albumin antibody (1:100) followed by a fluorescence labeled secondary antibody. Nuclei were counterstained with 4′, 6-diamidino-2-phenylindole (DAPI) and visualized with fluorescence microscopy or confocal laser scanning microscope.

### Histological and immunohistochemical analyses

Fresh liver samples were fixed in 10% formaldehyde and embedded in paraffin. 4 μm sections were stained with hematoxylin and eosin and sirus red using conventional protocols. For immunohistochemistry, samples were incubated with anti-α-SMA (1:10,000), anti-collagen I (1:200), anti-8-OHdG (1:100), anti-PGC-1α (1:300), or anti-Drp1 (1:200) primary antibodies overnight at 4 °C. Positive staining was visualized with 3,3′-diaminobenzidine (DAB). Human tissue arrays containing 8 normal liver tissues and 12 fibrotic liver tissues were purchased from Shanghai Zhuohan Biotechnology Co., Ltd (Shanghai, China). The protocol for human tissue was approved by the ethics committee of Capital Medical University. The specimens were independently diagnosed by two pathologists.

### Transmission electron microscopy

Fresh liver samples were processed for transmission electron microscopy (TEM). Samples were placed in 2.5% gluteraldehyde, washed three times in phosphate buffered saline (pH 7.4), and fixed in 1% osmium tetraoxide for 1 h at 4 °C. Samples were dehydrated in a graded series of ethanol solutions (50, 70, 90, and 100% for 20 min each), embedded in epoxy resin, and cut into ultrathin sections. Sections were mounted on copper grids, stained with uranyl acetate and lead citrate, and observed under a transmission electron microscope.

### Statistical analysis

Statistical analyses were conducted with the SPSS 12.0 software. GraphPad was used to export graphs. Variables are expressed as mean±standard deviation (SD). Comparisons were evaluated with the two-tailed Student’s *t* test and ANOVA. *P* < 0.05 was considered statistically significant.

## Results

### Liver fibrosis was effectively reversed by ROS scavengers

A mouse model of liver fibrosis was established after 4 weeks of CCl_4_ treatment. Previous studies have shown that oxidative stress has a key role in the process of liver fibrosis and may directly cause hepatocyte degeneration and necrosis^23,24^. Accordingly, in liver tissue derived from mice challenged with CCl_4_ for 8 weeks, there were significant increases in 3-nitrotyrosine (a biomarker of oxidative damage), collagen I, and α-SMA protein levels compared to controls. Treatment with the ROS scavengers apocynin and mito-tempo effectively attenuated the CCl_4_-induced increases in 3′-nitrotyrosine, collagen I, and α-SMA protein levels (Fig. 1b, c) and markedly decreased the number of fibrous septa in liver tissue (Fig. 1b). These results suggest that inhibition of oxidative stress with ROS scavengers can suppress liver fibrogenesis.

### Oxidative stress-induced mitochondrial DNA damage and depletion were associated with an imbalance in mitochondrial dynamics in the fibrotic liver

**Fig. 2:**
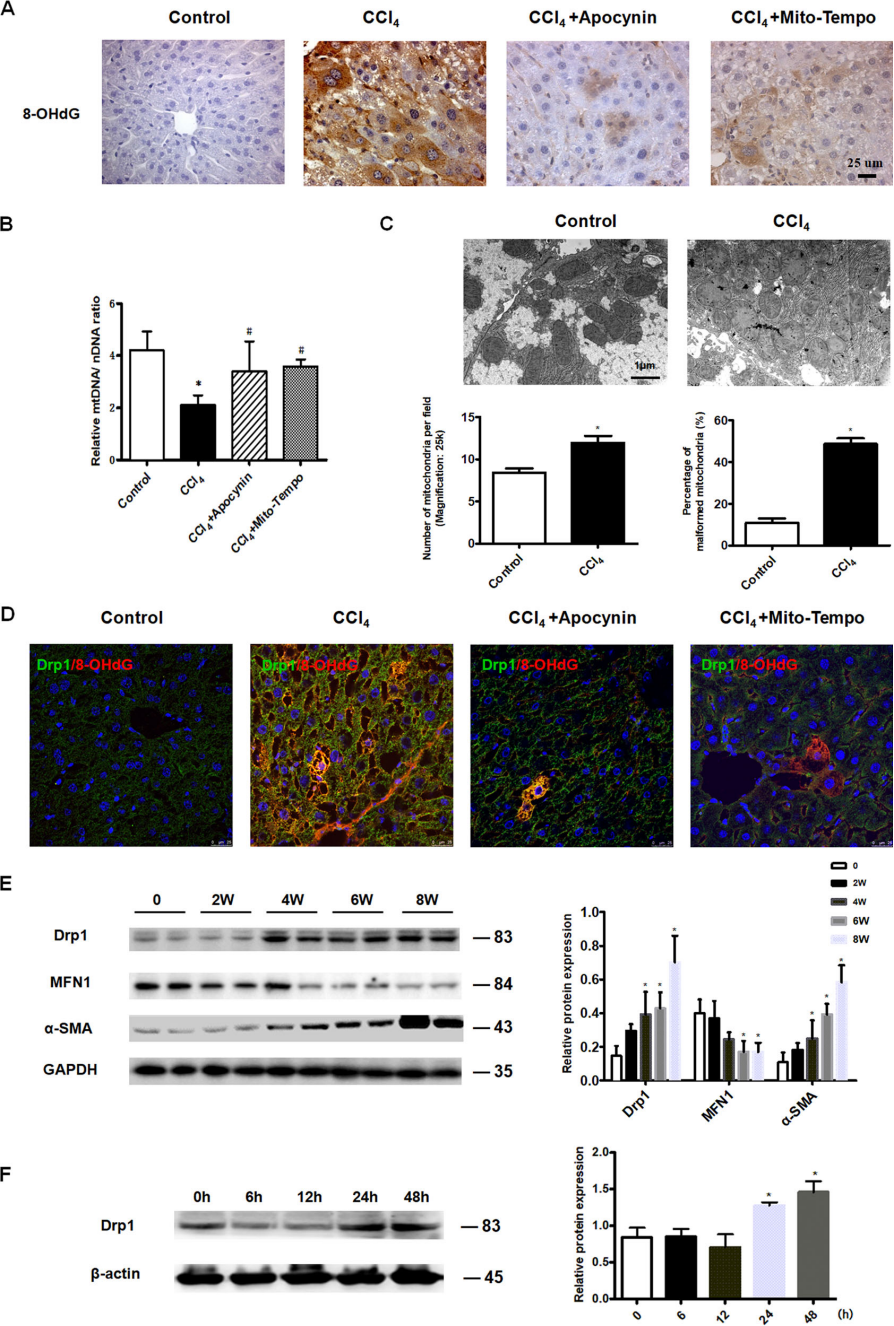
Oxidative stress increased hepatocyte mitochondrial fission. **a** Immunohistochemical staining showing an increase in 8-OHdG expression and damage to mitochondrial DNA in liver tissue derived from mice treated with CCl_4_ for 8 weeks compared to controls. Treatment with apocynin or mito-tempo attenuated the CCl_4_-induced increase in 8-OHdG (40×). **b** Mitochondrial DNA copy number was decreased in liver tissue derived from mice treated with CCl_4_ for 8 weeks, an effect that was reversed by apocynin or mito-tempo treatment. *P < 0.01 vs. control, ^#^*P* < 0.05 vs. CCl_4_ group. **c** Transmission electron microscopy showing hepatocyte mitochondria in CCl_4_-treated and control mice. Mitochondrial number and the percentage of malformed mitochondria were increased in hepatocytes derived from CCl_4_-treated compared to control mice, *P < 0.05 vs. control. **d** Immunofluorescence staining showing Drp1 and 8-OHdG expression was increased in liver tissue derived from mice treated with CCl_4_ for 8weeks; treatment with apocynin or mito-tempo attenuated the CCl_4_-induced increase in Drp1 and 8-OHdG. Green, Drp1; red, 8-OHdG (63×). **e** Western blot analysis showing Drp1, MFN1 and α-SMA protein levels in liver tissue derived from mice treated with CCl_4_ for 0, 2, 4, 6 and 8 weeks, *P < 0.05 vs. control. For (**a**–**e**), *n* = 4 mice in each group; experiments were repeated at least three times. The total number of animals in each group was approximately 12. **f** Western blot analysis showing Drp1 protein levels were increased in L-02 cells treated with 300 μM H_2_O_2_. β-actin served as the internal control. *P < 0.05 vs. 0 h, data represent at least four independent treatments.

Oxidative stress is an important cause of mitochondrial dysfunction and has a role in the pathogenesis of liver disease^25^. We explored whether oxidative stress increased hepatocyte mitochondrial DNA damage in liver fibrosis. In liver tissue derived from mice challenged with CCl_4_ for 8 weeks, there was an obvious increase in 8-OHdG (a marker of oxidative stress to DNA) expression compared to controls (Fig. 2a). Treatment with the ROS scavengers apocynin and mito-tempo effectively attenuated the CCl_4_-induced increase in 8-OHdG expression, suggesting that ROS were a key factor in hepatocyte mitochondrial DNA damage in liver fibrosis (Fig. 2a). Furthermore, CCl_4_ treatment was associated with decreases in mitochondrial DNA copy number in mouse liver tissue compared to controls, indicating mitochondrial DNA depletion, an effect that was reversed by the ROS scavengers apocynin and mito-tempo (Fig. 2b).

To counteract oxidative stress-induced-hepatocyte mitochondrial DNA damage in the fibrotic liver, Drp1 (mitochondrial fission protein) protein levels were increased (Fig. 2d, e), MFN1 (mitofusin-1, a protein that is a mediator of mitochondrial fusion) protein levels were decreased (Fig. 2e). Consequently, mitochondrial number was increased; however, most of the mitochondria in the fibrotic hepatocytes were malformed, appearing as small globules or balloon-shapes with few cristae (Fig. 2c). Mitochondrial damage and excessive fission were effectively inhibited by the ROS scavengers apocynin and mito-tempo (Fig. 2d). These results suggest that mitochondrial dynamics were altered in the fibrotic liver. To further determine whether abnormal mitochondrial fission was caused by oxidative stress, L-02 cells were treated with H_2_O_2_ for 24 h, after which there was an obvious increase in Drp1 protein levels (Fig. 2f). These results suggest that oxidative stress can lead to hepatocyte mitochondrial damage and excessive fission in the fibrotic liver.

### Excessive mitochondrial fission was associated with hepatocyte EMT and liver fibrosis

**Fig. 3:**
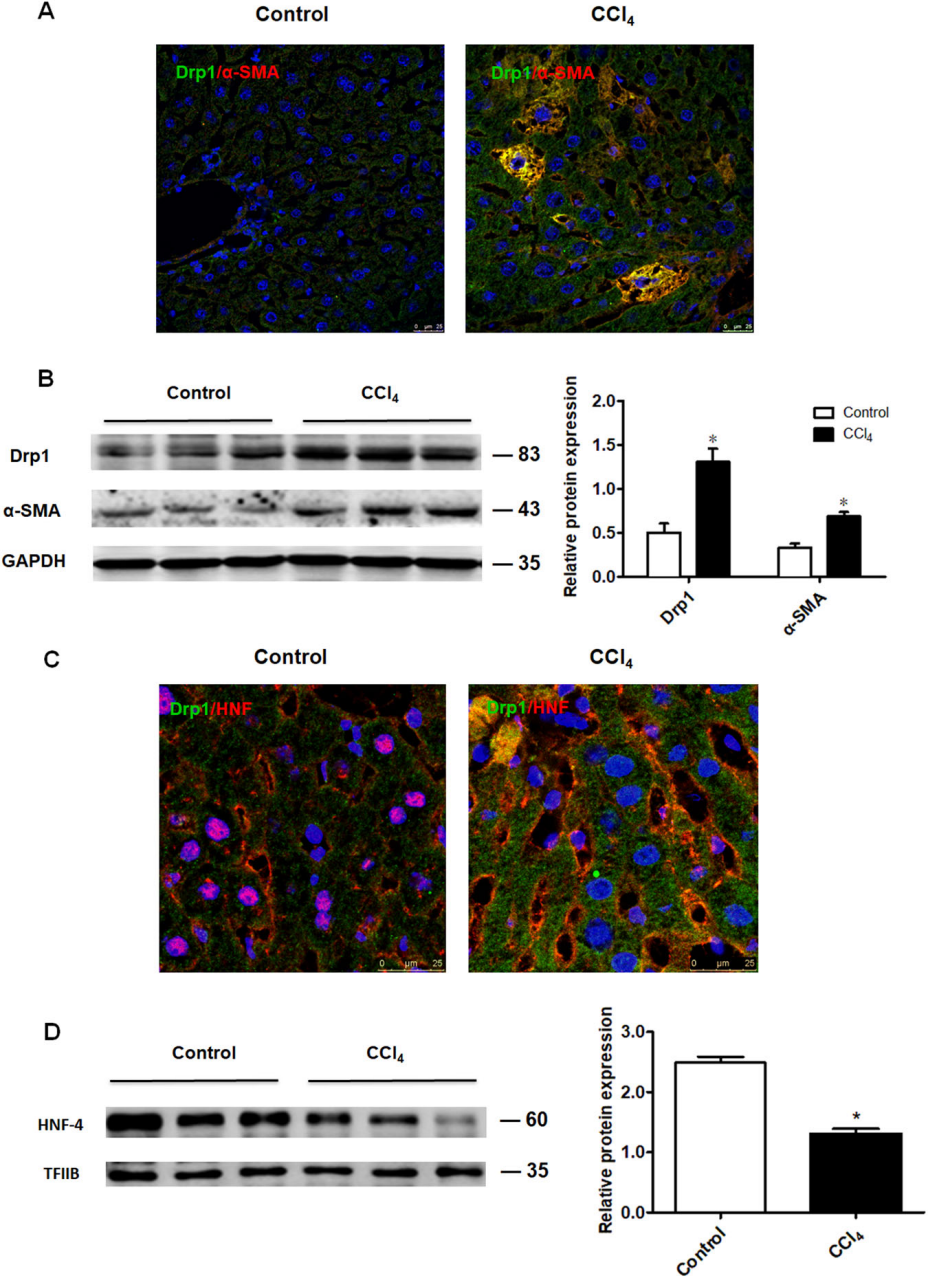
Excessive mitochondrial division was associated with hepatocyte EMT in liver tissue derived from mice treated with CCl_4_ for 6 weeks. **a**, **c** Immunofluorescence staining showing Drp1 and α-SMA expression (**a**) (40×) and Drp1 and HNF-4 expression (**c**) (63×) in the control and fibrotic liver. Green, Drp1; red, α-SMA and HNF-4. **b**, **d** Western blot analysis showing Drp1, α-SMA, and HNF-4 protein levels in the control and fibrotic liver. GAPDH served as an internal control for Drp1 and α-SMA; TFIIB served as internal control for HNF-4; bands represent three individual mice; *n* = 4 mice in each group; experiments were repeated at least three times. The total number of animals in each group was approximately 12. *P < 0.05 vs. control.

To determine whether excessive mitochondrial fission caused by oxidative stress could induce hepatocyte EMT, α-SMA (mesenchymal marker), HNF-4 (hepatocyte marker), and Drp1 protein levels were detected in liver tissue derived from mice treated with CCl_4_ and controls. In liver tissue derived from mice challenged with CCl_4_ for 6 weeks, Drp1 and α-SMA protein expression were increased (Fig. 3a, b) and some Drp1 positive hepatocytes were α-SMA positive (Fig. 3a), indicating the association between abnormal mitochondrial fission and the hepatocyte mesenchymal phenotype. Further, some Drp1 positive hepatocytes were HNF-4 negative (Fig. 3c) and HNF-4 protein levels were decreased (Fig. 3d), indicating that excessive mitochondrial fission may promote hepatocytes to lose their epithelial phenotype in the fibrotic liver. These results suggest that excessive mitochondrial fission caused by oxidative stress plays a role in liver fibrosis.

### Inhibition of mitochondrial fission suppressed hepatocyte EMT and liver fibrosis

**Fig. 4:**
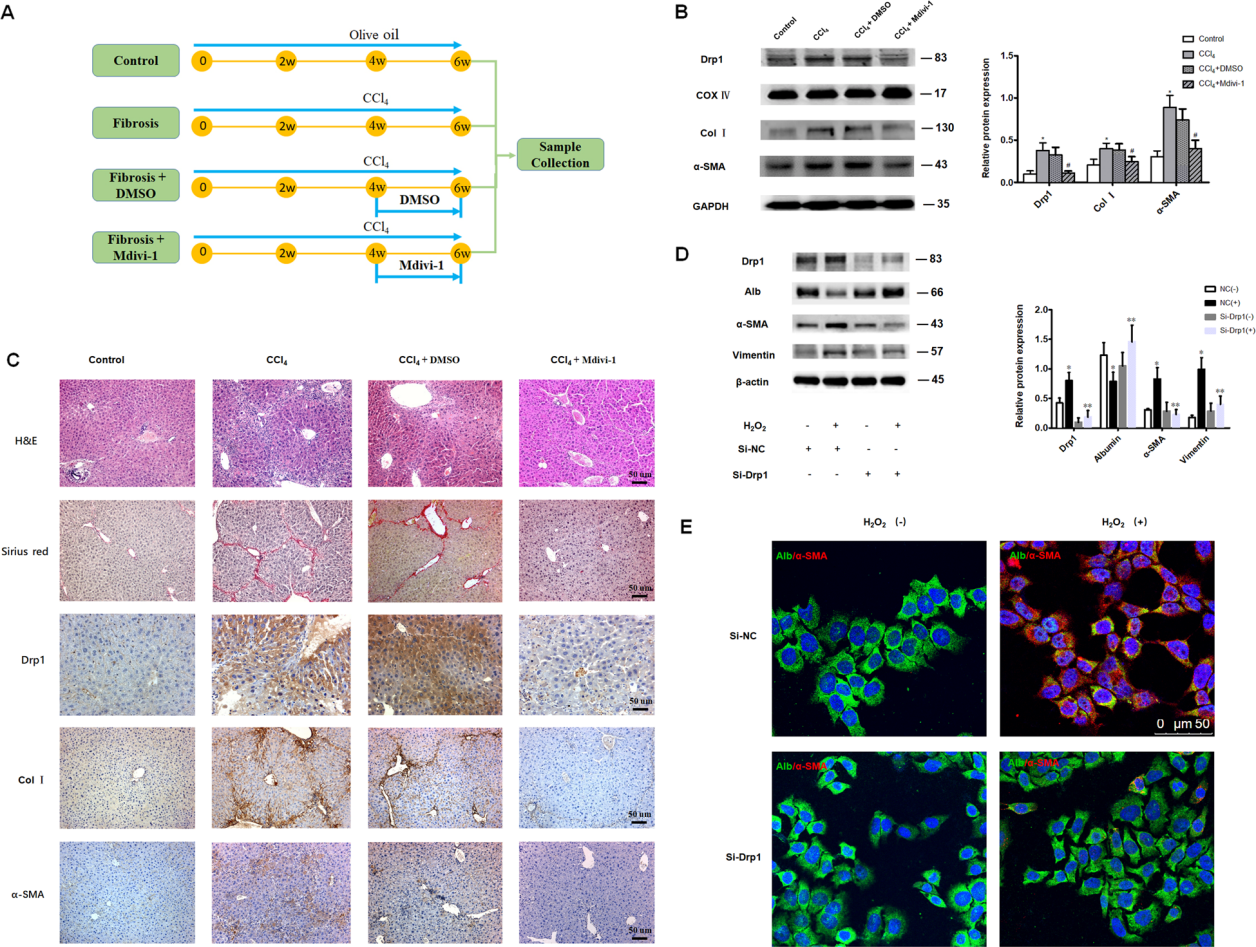
Inhibition of Drp-1 suppressed hepatocyte EMT and liver fibrosis. **a** Mdivi-1 treatment protocol. **b** Western blot analysis showing Drp1, collagen I and α-SMA protein levels in liver tissue derived from mice treated with control, CCl_4_, CCl_4_ + DMSO and CCl_4_ + Mdivi-1, COX IV served as the internal control for mitochondrial protein and GAPDH served as the internal control for collagen I and α-SMA. **c** Hematoxylin and eosin, sirius red and immunohistochemical staining showing Drp-1, collagen I and α-SMA expression in liver tissue derived from mice treated with control, CCl_4_, CCl_4_ + DMSO and CCl_4_ + Mdivi-1. Col I: Collagen I (X20). For **b**–**c**, *n* = 4 mice in each group; the experiments were repeated at least three times. The total number of animals in each group was approximately 12. *P < 0.05 vs. control, ^#^*P* < 0.05 vs. CCl_4_ group. (**d**,#**e**) L-02 cells were transfected with Drp1 siRNA or NC siRNA for 8 h and challenged with 300 uM H_2_O_2_ (+) or remained untreated (−) for 48 h before Western blot analysis (**d**) or immunofluorescence staining (**e**), Alb: Albumin, Green; α-SMA, red (40×). **d** Transfection with Drp1 siRNA attenuated H_2_O_2_-induced increases in vimentin and α-SMA protein levels, and maintained albumin protein level. Data were from 4 independent transfection experiments, *P < 0.05 vs. NC siRNA (−), **P < 0.05 vs. NC siRNA (+). β–actin served as the internal control.

We determined whether inhibition of mitochondrial fission suppressed hepatocyte EMT and alleviated liver fibrosis in mice. Drp1 protein levels in mitochondrial protein isolated from the liver of fibrotic mice treated with Mdivi-1, a selective Drp1 inhibitor, were significantly decreased compared to mice challenged with CCl_4_ alone (Fig. 4b). Further, Mdivi-1 alleviated histopathological changes in the fibrotic liver by decreasing collagen I and α-SMA expression (Fig. 4c).

To further clarify the role of Drp1 in liver fibrosis, L-02 cells were transfected with Drp1 siRNA and treated with H_2_O_2_. Protein levels of Drp1 and the mesenchymal markers α-SMA and vimentin were significantly decreased but albumin protein levels were maintained in H_2_O_2_-treated L-02 cells transfected with Drp1 siRNA compared to H_2_O_2_-treated L-02 cells transfected with NC siRNA (Fig. 4d, e). These results suggest that Drp1 is involved in hepatocyte EMT, and inhibition of Drp1 may suppress hepatocyte EMT and effectively relieve liver fibrosis.

### Decreased PGC-1α expression was associated with hepatic fibrosis

**Fig. 5:**
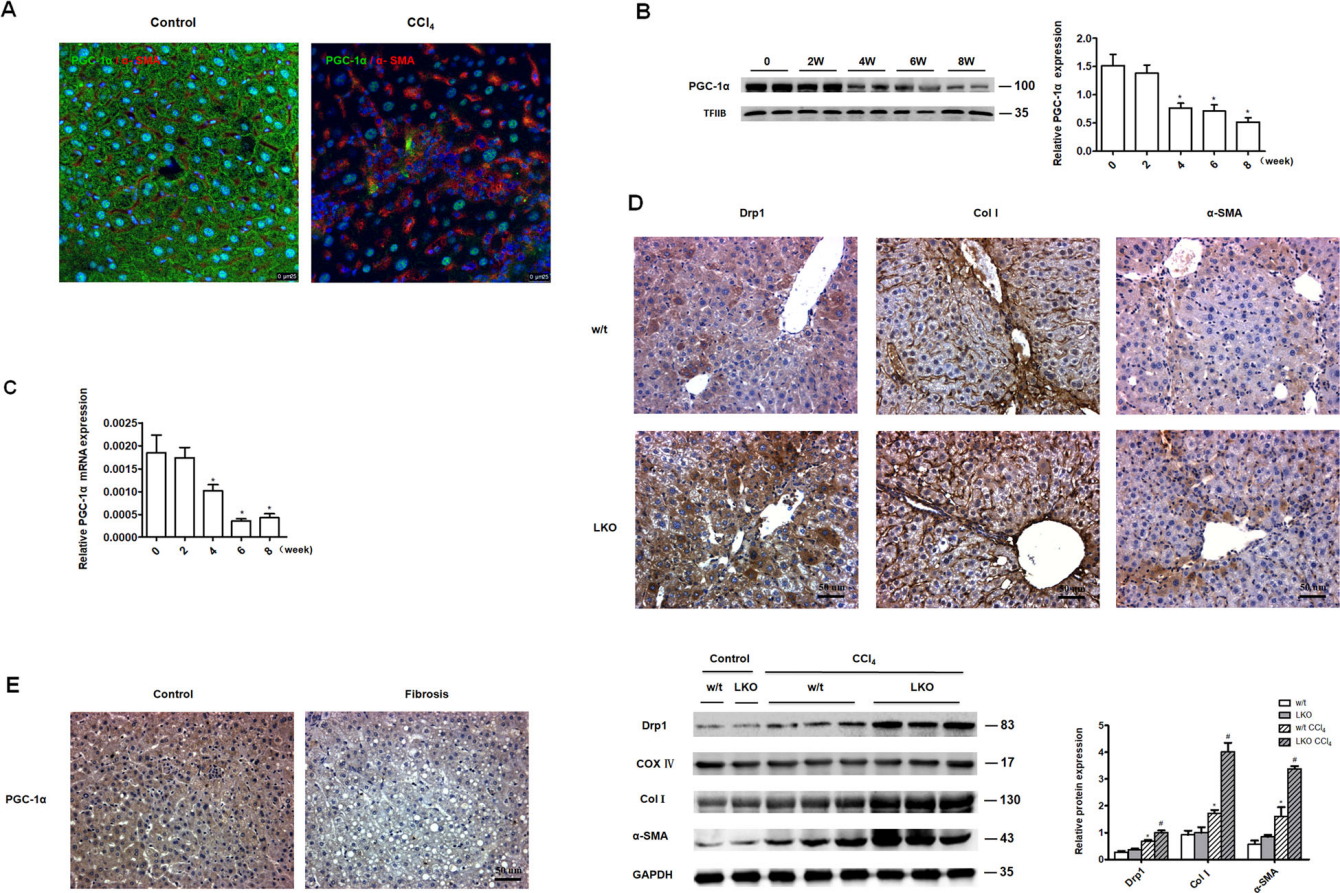
Decreased PGC-1α expression was associated with hepatic fibrosis. **a** Immunofluorescence staining showing PGC-1α and α-SMA expression. Green, PGC-1α; red, α-SMA (63×). **b** Western blot analysis showing PGC-1α levels in liver nuclear protein derived from mice treated with CCl_4_, *P < 0.05 vs. 0 weeks. TFIIB served as internal control for PGC-1α. **c** Quantitative PCR showing PGC-1α mRNA levels in liver tissue derived from mice treated with CCl_4_, *P < 0.05 vs. 0 weeks. **d** Immunohistochemical staining (20×) and representative western blot analysis showing Drp1, collagen I and α-SMA protein levels in liver tissue derived from wild type and hepatocyte-specific PGC-1α^f/f^ alb cre^+/0^ mice treated with CCl_4_ for 6 weeks, *P < 0.05 vs. w/t control, ^#^*P* < 0.05 vs. w/t CCl_4_ group. Cox IV served as the internal control for liver mitochondrial protein and GAPDH served as the internal control for collagen I and α-SMA. w/t: wild type mice; LKO: hepatocyte-specific PGC-1α^f/f^ alb cre^+/0^ mice. For (**a**–**d**), *n* = 4 mice in each group; the experiments were repeated at least three times. The total number of animals in each group was approximately 12. **e** Immunohistochemistry staining showing PGC-1α expression in normal human liver tissues (*n* = 8) and fibrotic liver tissues (*n* = 12) (20×).

PGC-1α, a key regulator of mitochondrial biogenesis, is mainly expressed in tissues with high energy demands that are rich in mitochondria, such as heart, kidney, skeletal muscle and liver^26^. In the present study, PGC-1α protein was present in hepatocytes in liver tissue derived from control mice. In liver tissue derived from mice challenged with CCl_4_ for 6 weeks, PGC-1α expression was obviously decreased and α-SMA expression was obviously increased compared to controls (Fig. 5a). There was a progressive decrease in PGC-1α levels with increasing duration of CCl_4_ treatment (Fig. 5b, c). In liver tissue derived from hepatocyte-specific PGC-1α knockout mice treated with CCl_4_ for 6 weeks, more extensive fibrosis, as evidenced by increased collagen I and α-SMA protein levels, was associated with increased Drp1 expression than in liver tissue derived from CCl_4_-treated wild type mice (Fig. 5d), indicating that regulation of mitochondrial dynamics by PGC-1α contributes to liver fibrogenesis. In human tissue, PGC-1α protein levels appeared lower in fibrotic liver compared to controls (Fig. 5e). These results suggest a potential protective role for PGC-1α in liver fibrosis.

### Oxidative stress-induced PGC-1α down regulation led to abnormal mitochondrial fission and hepatocyte EMT

**Fig. 6:**
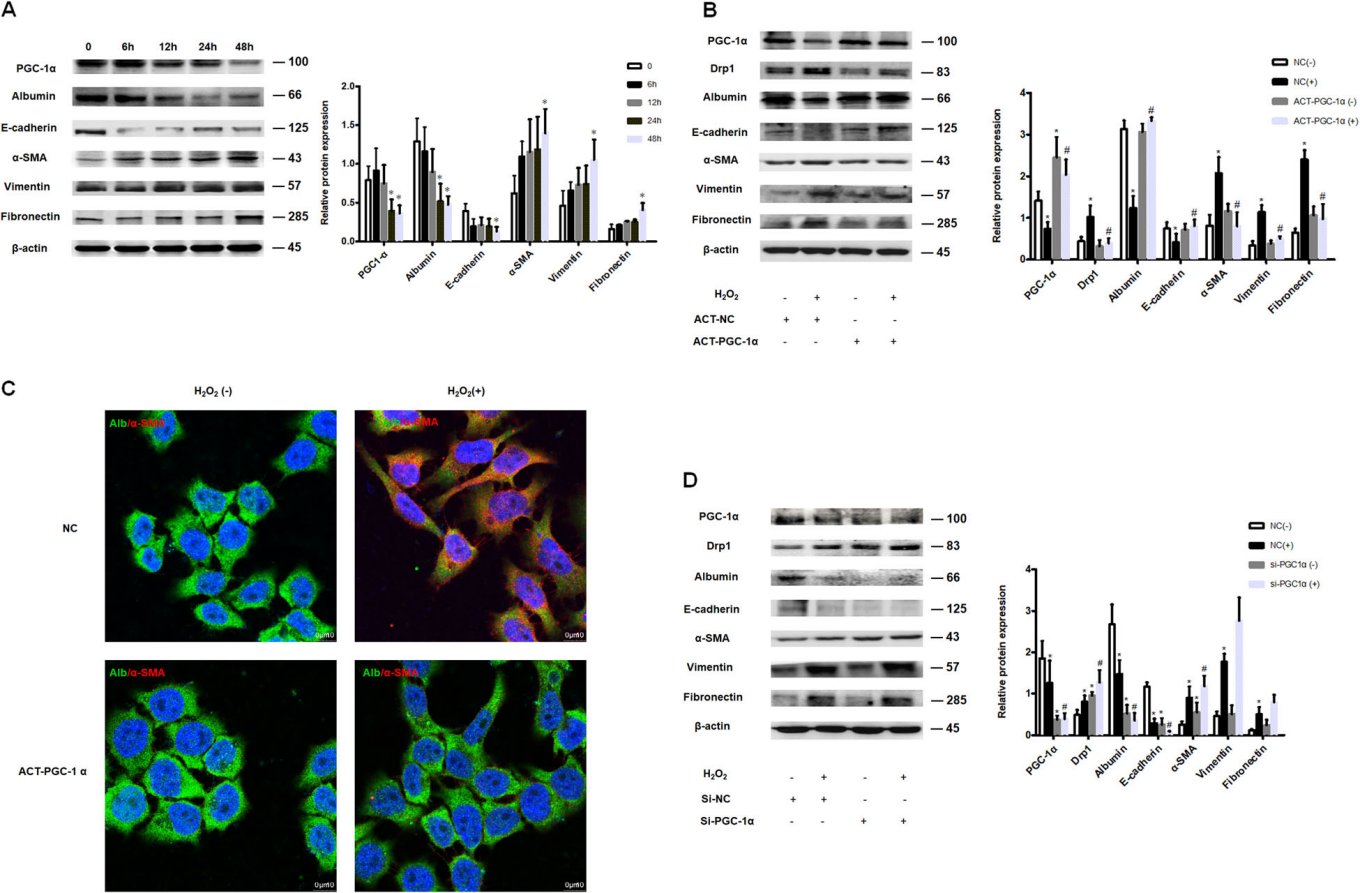
Oxidative stress-induced PGC-1α down regulation led to abnormal mitochondrial fission and hepatocyte EMT. **a** Western blot analysis showing PGC-1α, albumin, e-cadherin, α-SMA, vimentin and fibronectin protein levels in L-02 cells treated with 300 μM H_2_O_2_ for up to 48 h. β-actin was used as loading control. Data represent 4 independent H_2_O_2_ treatments, *P < 0.05 vs. 0 h. **b**, **d** Western blot analysis showing overexpression of PGC-1α attenuated hepatocyte EMT while inhibition of PGC-1α accelerated hepatocyte EMT. L-02 cells were transfected with PGC-1α activation plasmid (**b**) or PGC-1α siRNA (**d**), or control and treated with 300 μM H_2_O_2_ (+) or untreated (−) for 48 h. PGC-1α, Drp1, albumin, e-cadherin, α-SMA, vimentin and fibronectin protein levels were examined. β –actin served as the internal control. Data represent 4 independent transfections, *P < 0.05 vs. NC (−), ^#^P < 0.05 vs. NC (+). **c** Immunofluorescence staining showing albumin and α-SMA in L-02 cells transfected with human PGC-1α activation plasmid or control and treated with 300 µM H_2_O_2_ (+) or untreated (−) for 48 h. Alb: Albumin, Green; α-SMA, red (63×).

To evaluate whether oxidative stress-induced mitochondrial fission was associated with decreased expression of PGC-1α, L-02 cells were treated with H_2_O_2_ for up to 48 h. PGC-1α protein level was decreased and Drp1 protein level was increased in H_2_O_2-_treated cells compared to controls (Figs. 2f, 6a). Furthermore, after H_2_O_2_ treatment, L-02 cells lost their epithelial phenotype and assumed a mesenchymal phenotype, characterized by a decrease in albumin and e-cadherin protein levels and an increase in α-SMA, vimentin and fibronectin protein levels (Fig. 6a). To further explore the role of PGC-1α in protecting hepatocyte mitochondria from fission and hepatocytes from subsequent EMT, a PGC-1α activation plasmid was transfected to H_2_O_2_ treated and control L-02 cells. PGC-1α overexpression markedly reduced Drp1 protein levels, repressed the H_2_O_2_-induced increase in α-SMA, vimentin, and fibronectin protein levels, and maintained albumin and e-cadherin protein levels, suggesting that PGC-1α overexpression prevented hepatocyte EMT (Fig. 6b, c). Conversely, transfection with PGC-1α siRNA significantly increased Drp1 and EMT in H_2_O_2_ treated L-02 cells (Fig. 6d).

## Discussion

Liver fibrosis results from the repair of chronic liver injuries and eventually leads to cirrhosis and end stage liver disease. The fibrotic response involves over deposition of ECM proteins in the liver^27^ and reflects a balance between ECM production and degradation, such that fibrosis is reversible in its initial stages^28^. There are several cellular sources of ECM in the liver, including hepatic stellate cells (HSC), portal fibroblasts, bone marrow-derived cells, and fibroblasts derived from hepatocyte EMT^29–32^.

Mitochondria are important organelles, which are often referred to as the ‘power stations’ of eukaryotic cells. Approximately 90% of ATP is generated by mitochondria through oxidative phosphorylation, which is defined as an electron transport chain driven by substrate oxidation^33^. ROS act as an important intracellular messenger, activating multiple signal transduction pathways; although, overproduction of ROS can cause tissue and cell injury^34^. Oxidative stress results in lipid peroxidation, intracellular protein and enzyme degeneration, and oxidative DNA damage^35,36^. Mitochondria are particularly susceptible to oxidative stress. We hypothesized that oxidative stress-induced mitochondrial damage in hepatocytes plays an important role in the process of liver fibrosis. The present study assessed the role of mitochondrial dynamics in hepatocyte EMT and an in vitro human (L-02 cells) hepatic cell line and an in vivo mouse model of liver fibrosis. Findings showed that oxidative stress caused mitochondrial DNA damage, evidenced by the expression of 8-OHdG protein in the cytoplasm of hepatocytes and mitochondrial DNA depletion in mice, while a scavenger of ROS alleviated mitochondrial damage and liver fibrosis. Mitochondrial mass was increased in the mouse fibrotic liver compared to controls; however, most of the mitochondria were fragmented, which was likely a function of increased fission.

Mitochondria are dynamic organelles, frequently dividing and fusing with each other. Mitochondrial fission is regulated by Drp1. Evidence suggests that Drp1 activation is dependent on ROS, ERK, and JNK^37,38^. ROS lead to Drp1 phosphorylation at the Ser616 site, which promotes migration of Drp1 from the cytosol to the mitochondrial surface and triggers mitochondrial fission^39^. In the present study, Drp1 was increased in mitochondrial protein isolated from mouse fibrotic liver and in H_2_O_2_-treated L-02 cells compared to controls. These findings indicate that oxidative stress-induced recruitment of Drp1 to the mitochondrial surface and increased mitochondrial fission. Some Drp1 positive hepatocytes were α-SMA positive but HNF-4 negative, suggesting Drp1 overexpression was associated with hepatocyte EMT. To further investigate this, mice challenged with CCl_4_ were treated with Mdivi-1, a cell-permeable selective inhibitor of mitochondrial division, to determine if inhibition of mitochondrial fission could reverse liver fibrosis. Mdivi-1 decreased the number of α-SMA positive hepatocytes and alleviated fibrosis. In vitro siDrp1 transfection in L-02 cells confirmed that inhibition of Drp1 attenuated hepatocyte EMT. Similarly, a decrease in mitochondrial fission significantly diminished liver injury and fibrosis in in vitro and in vivo mouse models of bile acid-induced liver injury^40^.

We explored the factors responsible for regulating mitochondrial fission in the fibrotic liver. PGC-1α plays a crucial role in the regulation of mitochondrial biogenesis by coordinating the activity of nuclear respiratory factors (NRFs), activating genes involved in the respiratory chain and fatty acid oxidation (FAO), increasing mitochondrial number, and augmenting the respiratory capacity of mitochondria^41–43^. In the present study, PGC-1α expression was decreased in liver derived from mice challenged with CCl_4_ and humans with fibrosis. Interestingly, PGC-1α negative hepatocytes were positive for α-SMA, indicating that PGC-1α might play a role in liver fibrosis. To test this hypothesis, hepatocyte-specific PGC-1α knockout mice were treated with CCl_4_ for 6 weeks. Collagen I and α-SMA expression were increased in hepatocytes derived from CCl_4_-treated PGC-1α knockout mice compared to wild type mice. These data indicate that knockout for PGC-1α exacerbated liver fibrosis. In vitro studies confirmed that PGC-1α was decreased in H_2_O_2_-induced L-02 EMT, and silencing of PGC-1α further aggravated hepatocyte EMT. Enhanced PGC-1α expression mitigated H_2_O_2_ induced Drp-1 overexpression and hepatocyte EMT.

In conclusion, these studies utilizing an in vitro human and an in vivo mouse model of liver fibrosis indicate that PGC-1α has a protective role in oxidative stress-induced-hepatocyte EMT and liver fibrosis. Further studies are needed to fully elucidate the role of mitochondrial dynamics in hepatocyte EMT and liver fibrosis and their potential as therapeutic targets.
